# Clinical outcomes of takotsubo syndrome in patients with cancer: a systematic review and meta-analysis

**DOI:** 10.3389/fcvm.2023.1244808

**Published:** 2023-09-29

**Authors:** Takumi Osawa, Kazuko Tajiri, Masaki Ieda, Tomoko Ishizu

**Affiliations:** ^1^ Department of Cardiology, Institute of Medicine, University of Tsukuba, Tsukuba, Japan; ^2^ Department of Cardiology, Tsukuba Medical Center Hospital, Tsukuba, Japan; ^3^ Department of Cardiology, National Cancer Center Hospital East, Kashiwa, Japan; ^4^ Tsukuba Life Science Innovation Program (T-LSI), School of Integrative and Global Majors (SIGMA), University of Tsukuba, Tsukuba, Japan

**Keywords:** stress cardiomyopathy, apical ballooning, broken heart syndrome, stress-induced cardiomyopathy, cardio-oncology, onco-cardiology, malignancy, oncology

## Abstract

**Background:**

Recent studies suggested a relationship between Takotsubo syndrome (TTS) and malignancy. However, clinical outcomes of TTS associated with cancer have not been assessed completely. This study was aimed to investigate the outcomes of patients with TTS and cancer.

**Methods:**

We performed a systematic review and meta-analysis to evaluate the clinical outcomes of TTS in patients with and without malignancy. We systematically reviewed and analyzed 14 studies (189,210 patients) published in PubMed and Cochrane Library databases until December 2022. The primary outcome was all-cause mortality at the longest follow-up.

**Results:**

The prevalence of current or previous malignancy in patients with TTS was 8.7% (16,461 patients). Patients with TTS and malignancy demonstrated a higher risk of mortality at the longest follow-up than those with TTS alone (odds ratio [OR], 2.41; 95% confidence interval [CI]; 1.95–2.98; *P* < 0.001). Moreover, cancer was significantly associated with an increased risk of in-hospital or 30-day mortality (OR 2.36; 95% CI, 1.67–3.33; *P* < 0.001), shock (OR 1.42; 95% CI, 1.30–1.55; *P* < 0.001), mechanical respiratory support (OR 1.68; 95% CI, 1.59–1.77; *P* < 0.001), arrhythmia (OR 1.27; 95% CI, 1.21–1.34; *P* < 0.001), and major adverse cardiac events (OR 1.69; 95% CI, 1.18–2.442; *P* < 0.001).

**Conclusions:**

This study revealed significant associations between previous or active cancer and an increased risk of all-cause mortality and in-hospital adverse events in patients with TTS.

## 1. Introduction

Takotsubo syndrome (TTS), also known as stress-induced cardiomyopathy or broken heart syndrome, is characterized by transient left ventricular systolic dysfunction without angiographic evidence of obstructive coronary artery disease. The prognosis does not appear to be as benign as previously reported, and the in-hospital mortality rate is as high as 5%. Moreover, some studies have reported long-term recurrence and increased mortality in patients with TTS (1, 2). Although TTS was reported to be associated with emotional or physical stress, the pathogenesis still remains unclear (3).

The common comorbidity associated with TTS is cancer. Previous studies reported a high incidence of malignancy in patients with TTS (4–6). Cancer; some anticancer drugs, including 5-fluorouracil, vascular endothelial growth factor inhibitors, immune checkpoint inhibitors; and the emotional and/or physiological stress associated with diagnosis, investigations, and treatment are recognized as triggers or predisposing factors for TTS (7–11). Recent observational studies have reported higher mortality in patients with TTS and malignancy compared to those with TTS alone (6, 12). Cancer may be an important prognostic factor in patients with TTS. Therefore, we conducted this meta-analysis to further investigate the association between TTS and cancer.

## 2. Methods

This meta-analysis was conducted according to the guidance from the Cochrane Handbook for Systematic Reviews and is reported according to the Preferred Reporting Items for Systematic Reviews and Meta-analyses (PRISMA) (13).

### 2.1. Study search and data sources

We performed an electronic search of the PubMed and Cochrane Library databases to identify all relevant articles related to TTS and cancer. We checked all records published from inception until December 18, 2022. We used the following search strategy: (“cancer” OR “carcinoma” OR “sarcoma” OR “melanoma” OR “cysts” OR “cyst” OR “neoplasms” OR “neoplasms” OR “neoplasm” OR “neurofibroma” OR “neurofibromas” OR “tumors” OR “tumoral” OR “tumorous” OR “tumour” OR “tumor” OR “tumours” OR “tumoural” OR “tumourous” OR “tumors” OR “malignancy” OR “oncology” OR “adenocarcinoma”) AND (“takotsubo” OR “stress cardiomyopathy” OR “stress-induced cardiomyopathy” OR “Takotsubo cardiomyopathy” OR “Tako tsubo cardiomyopathy” OR “Tako-tsubo cardiomyopathy” OR “apical ballooning syndrome” OR “transient apical ballooning syndrome” OR “broken heart syndrome” OR “Takotsubo syndrome” OR “left ventricular apical ballooning syndrome”). Further information was obtained through a manual search of references from recent reviews and relevant published original studies.

### 2.2. Selection criteria, data collection, and quality assessment

A systematic search was performed to identify retrospective and prospective cohort studies and case-control studies related to cancer and TTS. The exclusion criteria were as follows: (a) duplicate studies, (b) studies without usable data, (c) review articles, (d) case reports, (e) case series, (f) animal studies, and (g) non-English studies. Two investigators (T.O. and K.T.) screened all titles and abstracts independently. This was followed by full-text reviews of the selected articles by the same investigators.

The primary endpoint of this study was all-cause mortality at the longest follow-up. When mortality rates were reported for more than one observation period, the rate for the longer period was used. The secondary outcomes were in-hospital or 30-day mortality and in-hospital events such as cardiogenic shock, arrhythmia, major adverse cardiac events [MACE], cardiovascular death, and the need for mechanical respiratory or circulatory support.

### 2.3. Statistical analysis

Continuous data are expressed as mean ± standard deviation. The categorical data are presented as absolute numbers and percentages. All data analysis was performed using Stata 17.0 statistical software (Stata Corp, College Station, TX, USA) and Easy R based on R and R Commander (Jichi Medical University, Saitama, Japan) (14). Meta-analyses were conducted using a random-effect model. Pooled estimates were reported as odds ratios (ORs) and 95% confidence intervals (CIs) or as mean differences (MDs) and 95% CIs for continuous variables. Heterogeneity was evaluated using *I*^2^ statistics and *P*-values (low: 25%–50%, moderate: 50%–75%, and high: >75%). A forest plot was constructed using the same software. Publication bias was assessed using a funnel plot and Egger's test with a statistical cutoff of *P* = 0.1, and *P*-value <0.05 was considered statistically significant.

## 3. Results

### 3.1. Search results and characteristics of the included studies

**Figure 1:**
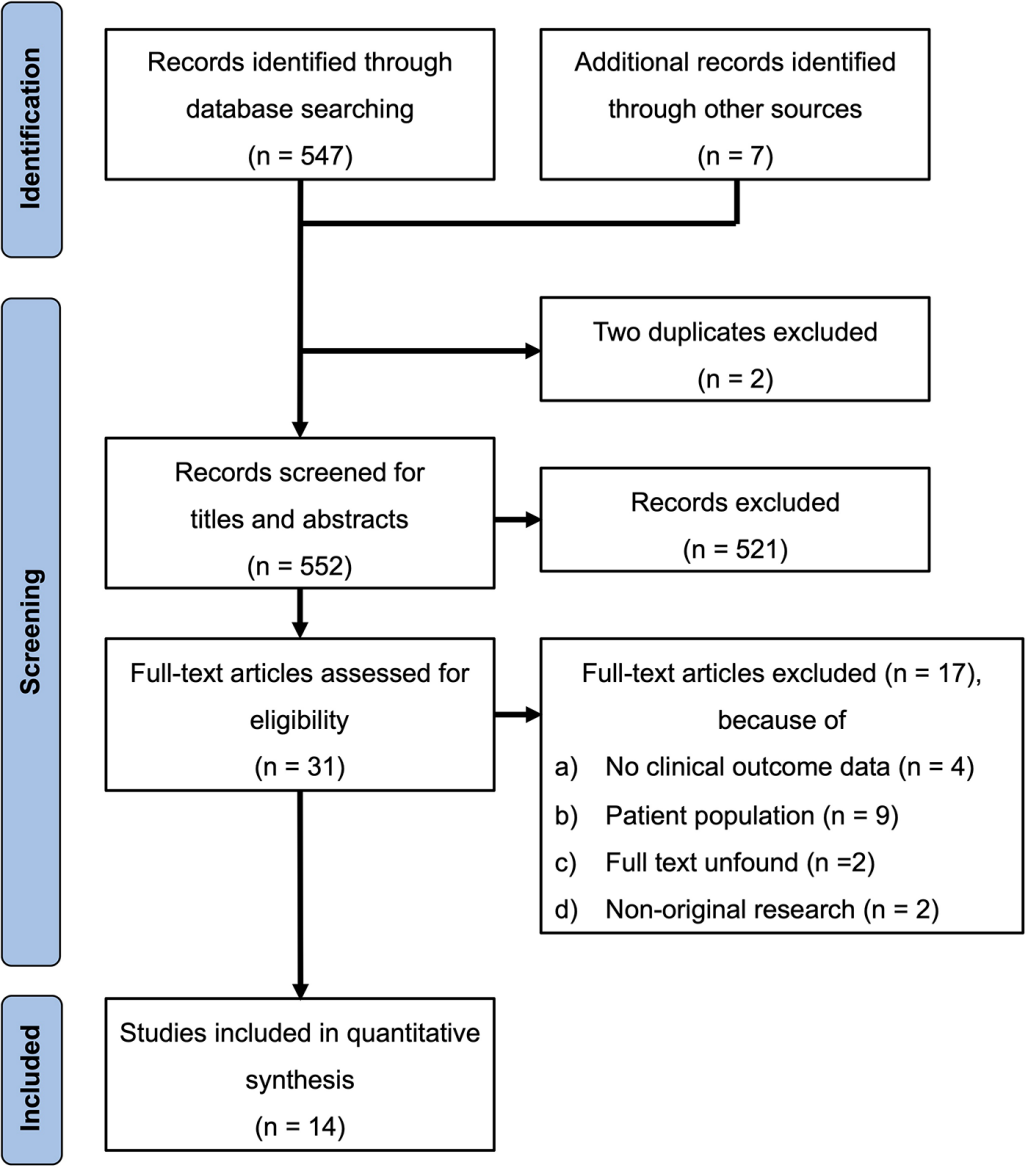
PRISMA flowchart of the study selection process. PRISMA, Preferred Reporting Items for Systematic Reviews and Meta-Analyses.

Figure 1 shows a flow diagram of data search and study selection. A total of 554 articles were found in the PubMed and Cochrane Library databases and by reviewing the bibliographies of relevant literature. After excluding two duplicates, 521 were excluded based on article titles and abstracts. After evaluating the full-text articles, 17 studies were excluded. Eventually, 14 studies with adequate data were included in our meta-analysis (4–6, 12, 15–24).

Finally, 189,210 patients with TTS were included. Of these, 16,461 (8.7%) reported malignancy. The baseline characteristics of the included studies are summarized in Table 1.

**Table 1:** Characteristics of the studies included in this meta-analysis.

Study	No. of patients	Patients with malignancy (%)	Most common type of malignancy	Study design	Follow-up duration
Pogran et al. (16)	147	25 (17%)	N/A	Retrospective	126 months
Tini et al. (15)	318	42 (13%)	Breast	Retrospective	2.7 (0.5–6.5) years
Jang et al. (17)	61,553	7,542 (12%)	N/A	Retrospective	Hospital admission days
Núñez-Gil et al. (6)	1,097	129 (12%)	Breast	Prospective	120 days
Cammann et al. (12)	1,604	267 (17%)	Breast	Prospective	10 years
Nguyen et al. (18)	346	58 (17%)	Breast	Prospective	4.1 (2.2–6.4) years
Zaghlol et al. (21)	318	81 (25%)	Breast	Retrospective	Hospital admission days
Joy et al. (20)	122,855	8,089 (7%)	N/A	Retrospective	Hospital admission days
Kim et al. (22)	265	70 (26%)	Breast	Prospective	5.8 ± 3.6 years
Möller et al. (19)	286	56 (20%)	Breast	Retrospective	4.0 ± 2.5 years
Sattler et al. (23)	114	25 (22%)	Colorectal cancer	Prospective	1,529 ± 1,121 days
Sobue et al. (24)	82	13 (16%)	N/A	Prospective	Hospital admission days
Girardey et al. (15)	154	44 (29%)	Digestive cancer	Retrospective	364 (79–878) days
Jo et al. (4)	71	20 (28%)	N/A	Prospective	Hospital admission days

### 3.2. Clinical characteristics of the patients with TTS with and without malignancy

TTS was reported in patients with cancer at an older age and was more common in males with cancer than in those without cancer (MD, 2.08; 95% CI, 0.31–3.85; *P* = 0.022; *I*^2 ^= 82%; *P* for heterogeneity <0.001 and OR, 0.64; 95% CI, 0.55–0.74; *P* < 0.001; *I*^2 ^= 65%; *P* for heterogeneity = 0.002, in patients with and without cancer, respectively) (Supplementary Figures 1, 2). Comorbidities (hypertension and diabetes) were similar among patients with TTS with and without malignancy (Supplementary Figures 3A,B). A physical trigger factor was more common in patients with cancer compared with the controls (OR, 1.75; 95% CI, 1.43–2.14; *P* < 0.001; *I*^2^ = 0%; *P* for heterogeneity = 0.425) (Supplementary Figure 4A), whereas emotional and unknown trigger factors were less frequent in patients with cancer (OR, 0.65; 95% CI, 0.47–0.90; *P* = 0.009; *I*^2 ^= 37%; *P* for heterogeneity = 0.209 and OR, 0.77; 95% CI, 0.61–0.97; *P* = 0.029; *I*^2 ^= 0%; *P* for heterogeneity = 0.309, respectively) (Supplementary Figures 4B,C). Symptoms of TTS differed significantly between patients with and without cancer. Moreover, studies reported more dyspnea (OR, 1.39; 95% CI, 1.15–1.68; *P* = 0.001; *I*^2 ^= 0%; *P* for heterogeneity = 0.570, Supplementary Figure 5A) but less chest pain (OR, 0.62; 95% CI, 0.51–0.76; *P* < 0.001; *I*^2 ^= 0%; *P* for heterogeneity = 0.960, Supplementary Figure 5B) in patients with cancer. Reduction in left ventricular ejection fraction at the onset of TTS was more in patients with cancer than in those without cancer (MD, −2.48; 95% CI, −3.52–−1.44; *P* < 0.001; *I*^2 ^= 0%; *P* for heterogeneity = 0.868, Supplementary Figure 6). In left ventricular ballooning patterns of TTS, the frequency of apical and basal types were similar among the patients with and without cancer (Supplementary Figures 7A,B), but the mid-ventricular type was more common in controls than in patients with cancer (OR, 0.56; 95% CI, 0.33–0.96; *P* = 0.036; *I*^2 ^= 0%; *P* for heterogeneity = 0.971, Supplementary Figure 7C).

**Figure 2:**
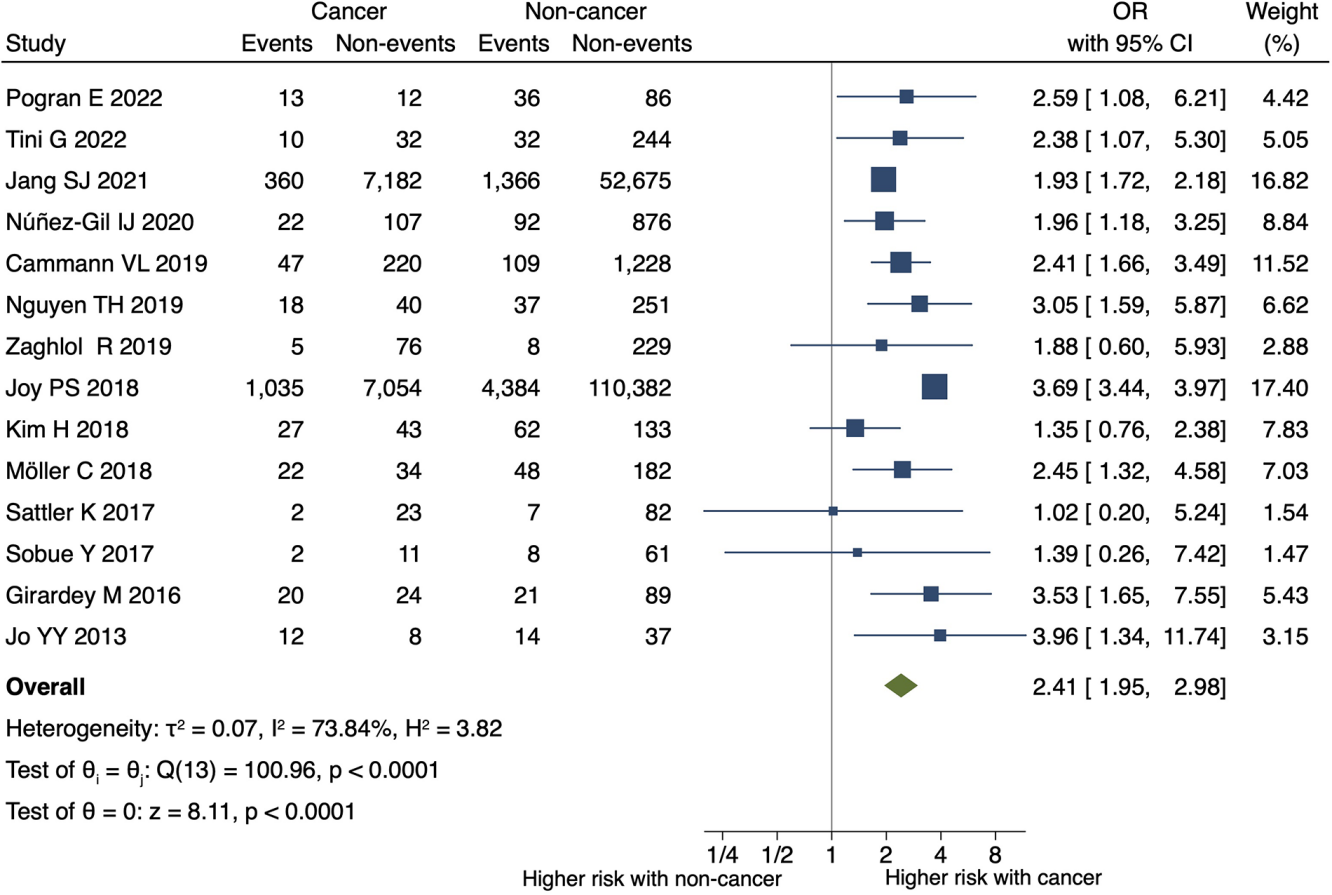
Impact of cancer on all-cause mortality at the longest follow-up in TTS.the forest plot shows the impact of cancer on all-cause mortality in TTS. CI, confidence interval; OR, odds ratio.

### 3.3. Outcomes in patients with TTS with and without malignancy

Patients with TTS and malignancy had a higher risk of all-cause mortality at the longest follow-up than the controls (OR, 2.41; 95% CI, 1.95–2.98; *P* < 0.001; *I*^2 ^= 74%; *P* for heterogeneity <0.001) (Figure 2). Supplementary Figure 8 shows a funnel plot of all-cause mortality. Egger's test detected no statistically significant publication bias (*P* = 0.433).

**Figure 3:**
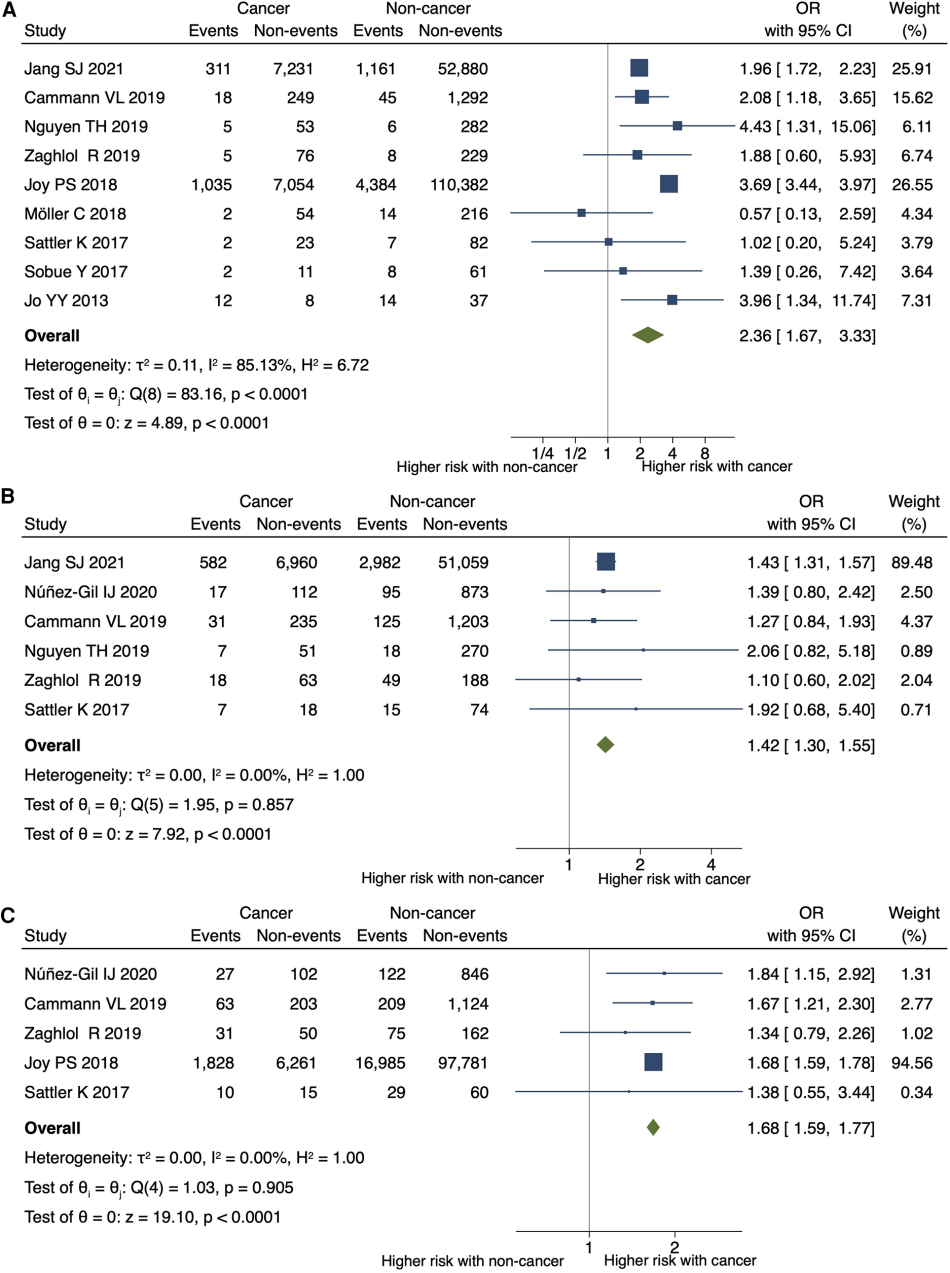
Impact of cancer on short-term mortality, shock, and respiratory support. Forest plots showing the impact of cancer on (**A**) in-hospital or 30-day mortality, (**B**) shock, (**C**) and the need for mechanical respiratory support. CI, confidence interval; OR, odds ratio.

**Figure 4:**
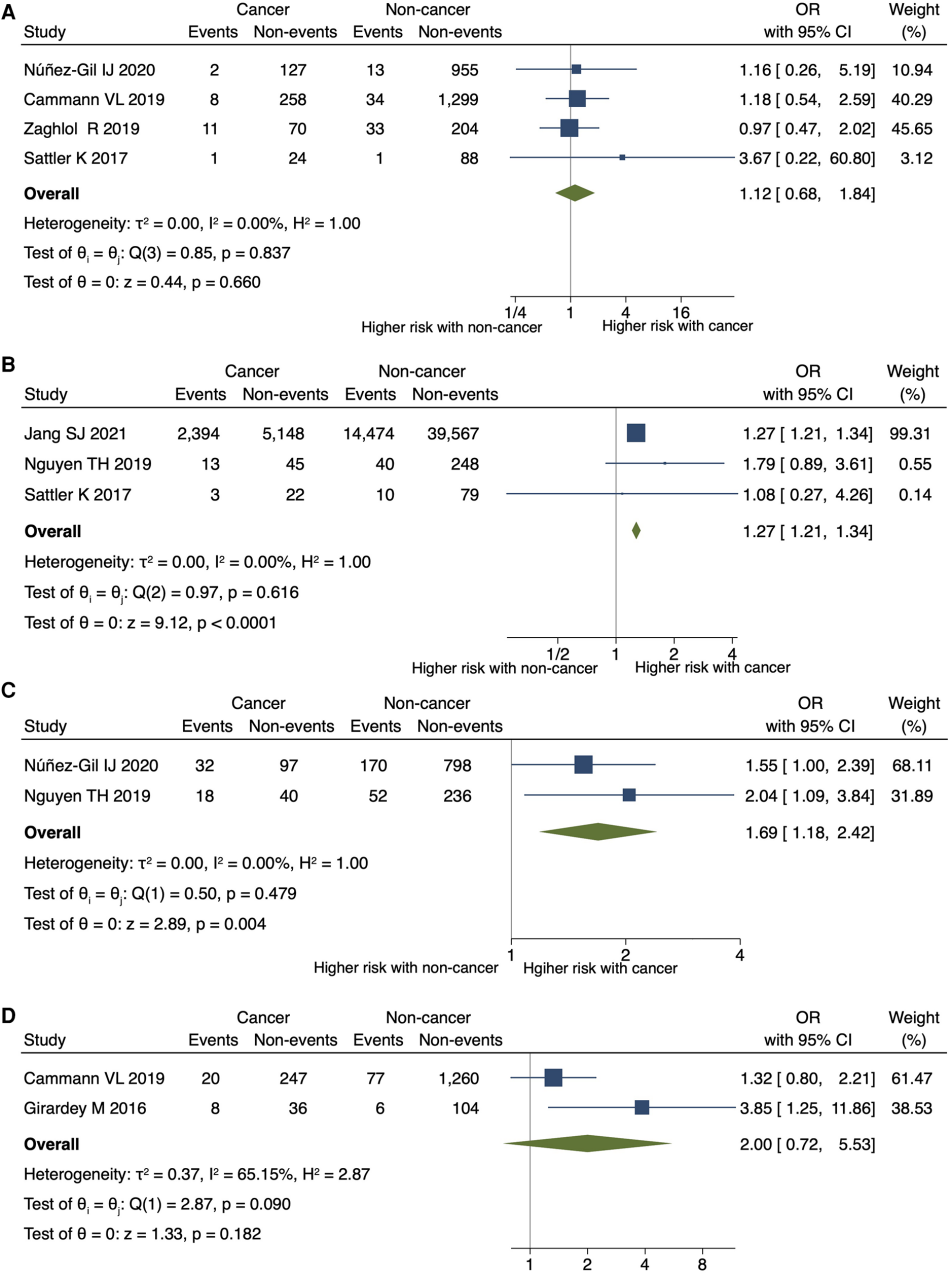
Impact of cancer on circulatory support, arrhythmia, MACE, and cardiovascular death. Forest plots showing the cancer impact on (**A**) the need for mechanical circulatory support, (**B**) arrhythmia, (**C**) MACEs, and (**D**) cardiovascular death. CI, confidence interval; MACEs, major adverse cardiac events; OR, odds ratio.

Cancer was significantly associated with an increased risk of in-hospital or 30-day mortality (OR 2.36; 95% CI, 1.67–3.33; *P* < 0.001; *I*^2 ^= 85%; *P* for heterogeneity <0.001) (Figure 3A), in-hospital shock (OR 1.42; 95% CI, 1.30–1.55; *P* < 0.001; *I*^2 ^= 0%; *P* for heterogeneity = 0.857), and the need for mechanical respiratory support (OR 1.68; 95% CI, 1.59–1.77; *P* < 0.001; *I*^2 ^= 0%; *P* for heterogeneity = 0.905) in patients with TTS (Figures 3B,C). No significant differences were observed in the need for mechanical circulatory support (OR 1.12; 95% CI, 0.68–1.84; *P* = 0.660; *I*^2 ^= 0%; *P* for heterogeneity = 0.837) (Figure 4A) between patients with and without cancer. In terms of cardiac events, patients with cancer had a significantly higher risk of arrhythmia and MACEs (Figures 4B,C) than those without cancer; however, the risk of cardiovascular death did not differ significantly between the two groups (Figure 4D).

## 4. Discussions

The key findings of our study elucidated that the co-existence of cancer was significantly associated with the following: (1) all-cause and short-term mortalities, (2) increased risk of shock, arrhythmia, and MACE, and 3) use of mechanical respiratory support in patients with TTS.

To the best of our knowledge, our study is the largest meta-analysis to investigate the association between cancer and TTS. Previously, three meta-analyses have reported the impact of cancer on patients with TTS (25–27). Meta-analyses by Brunetti et al. and Guo et al. reported poor in-hospital and follow-up clinical outcomes in patients with TTS complicated with cancer (25, 26). The meta-analysis by Genc et al. reported that short-term mortality is worse in patients with TTS complicated by malignancy (27). Our study confirmed these findings, and documented malignancy as a powerful predictor of all-cause and in-hospital deaths in patients with TTS. The present study demonstrated a 2.4-fold increase in all-cause and in-hospital mortalities in patients with malignancy and TTS. Recently, there have been significant advances in the diagnosis and treatment of both cancer and heart failure. Therefore, we conducted a new meta-analysis and found that the results are consistent with those reported in the previous meta-analyses. It is noteworthy that this study distinguishes itself by encompassing the largest cohort of both studies and patients when compared to the three preceding meta-analyses. Therefore, we re-examined in-hospital events other than death, which were not significant in previous studies, and found that arrhythmias and shock occurred more frequently in cancer patients than in noncancer patients during hospitalization.

Our meta-analysis showed that all-cause and short-term mortalities were significantly higher in patients with TTS complicated with cancer than in those without cancer. However, an increase in cardiovascular death in patients with TTS complicated by cancer was not statistically significant in this study. These findings suggest that noncardiac deaths account for a large proportion of all-cause mortality, and malignancy drives the outcomes of TTS. However, shock, arrhythmia, MACEs, and the need for mechanical respiratory support were more common in patients with cancer than in those without cancer. This might be because of the complex condition due to comorbidities in patients with cancer, and the development of TTS in patients with cancer might have worsened the condition. The incidence of in-hospital shock was significantly higher in patients with cancer, but the use of mechanical circulatory support was similar to that in patients without cancer. One possible reason for this may be that the introduction of mechanical circulatory support was postponed because of cancer. Currently, no guidelines exist for the diagnosis and treatment of TTS in patients with cancer. Understanding and assessing the clinical association between TTS and cancer are essential, which can help improve clinical outcomes. Discussions with a multidisciplinary team, including cardiologists and oncologists, are needed to treat these patients.

Interestingly, our study and other previous studies demonstrated that physical trigger factors were more common in cancer patients, while emotional and unknown trigger factors were less frequent in patients with cancer patients (25, 27). The triggers of TTS in patients with cancer encompass a spectrum of factors, including the presence of cancer itself, various psychological or physical triggers, including surgery, chemotherapy, radiation therapy, the diagnostic process, genetic predisposition, and acute complications like infections (28). The prevalence of psychiatric disorders has been reported to be not significantly different in patients with TTS with or without cancer (12). Physical pain from cancer, rather than psychological distress, could be considered a potential trigger for TTS. In patients with cancer undergoing chemotherapy or surgery, clinicians should maintain a high index of suspicion for TTS when assessing the sudden onset of cardiac dysfunction.

The pathophysiological mechanism between cancer and TTS remains unclear. There were multiple presumed mechanisms and associations have been reported. One of the proposed mechanisms was the activation of the sympathetic nervous system and coronary vasospasm (29). Specific chemotherapeutic agents have been implicated in TTS, including 5-fluorouracil, capecitabine, bevacizumab, combretastatin, rituximab, tyrosine kinase inhibitors, and immune checkpoint inhibitors (28).

This study had several limitations. First, this research was performed based on observational studies; therefore, the possibility of confounding biases cannot be ignored in this meta-analysis. Second, significant heterogeneity was found in the primary outcome. Third, the majority of the studies included in this meta-analysis did not present detailed information on laboratory tests, echocardiography, and angiography, making it difficult to discern whether TTS was correctly diagnosed. Fourth, the present study lacked information on the types, stages, and treatments of malignancy, and these cancer characteristics might have affected our results. Fifth, the diagnostic criteria for TTS differed among the included studies. It was not possible to ascertain whether heart disease other than takotsubo cardiomyopathy was excluded by catheterization or coronary CT scans.

## 5. Conclusions

Malignancy was associated with an increased risk of death and in-hospital adverse events in patients with TTS, and more careful follow-up is recommended for patients with TTS and cancer.

## Data Availability

The original contributions presented in the study are included in the article/**Supplementary Material**, further inquiries can be directed to the corresponding author.
