# Supplementary material for: Clinical outcomes of takotsubo syndrome in patients with cancer: a systematic review and meta-analysis
Source: Front Cardiovasc Med. 2023 Sep 29;10:1244808. doi: 10.3389/fcvm.2023.1244808 (PMC10570743; doi:10.3389/fcvm.2023.1244808)
Supplement: Supplementary file 1 [file Table1.docx]

Supplementary Material

Clinical Outcomes of Takotsubo Syndrome in Patients with Cancer: A Systematic Review and Meta-analysis

Takumi Osawa, Kazuko Tajiri^*^, Masaki Ieda, Tomoko Ishizu

*** Correspondence:** Kazuko Tajiri: ktajiri@east.ncc.go.jp

**Supplementary Figure 1.** **Forest plot showing the patients’ age in the cancer and non-cancer groups**

**Supplementary Figure 2.** **Forest plot showing the number of females in the cancer and non-cancer groups**

**Supplementary Figure 3.** **Forest plots showing the prevalence of (A) hypertension and (B) diabetes in the cancer and non-cancer groups**

**Supplementary Figure 4.** **Forest plots showing the prevalence of (A) physical, (B) emotional, and (C) unknown triggers in the cancer and non-cancer groups**

**Supplementary Figure 4.** **Forest plots showing the prevalence of (A) dyspnea and (B) chest pain in the cancer and non-cancer groups**

**Supplemental Figure 6. Forest plot showing the left ventricular ejection fraction at admission in the cancer and non-cancer groups**

**Supplemental Figure 7. Forest plots showing the prevalence of (A) apical, (B) basal, and (C) mid-ventricular patterns in the cancer and non-cancer groups**

**Supplemental Figure 8. Funnel plot showing publication bias of the included studies**
